# Cognitive representations of intracranial self-stimulation of midbrain dopamine neurons depend on stimulation frequency

**DOI:** 10.1038/s41593-024-01643-1

**Published:** 2024-05-13

**Authors:** Samuel J. Millard, Ivy B. Hoang, Savannah Sherwood, Masakazu Taira, Vanessa Reyes, Zara Greer, Shayna L. O’Connor, Kate M. Wassum, Morgan H. James, David J. Barker, Melissa J. Sharpe

**Affiliations:** 1 Department of Psychology, University of California, Los Angeles, Los Angeles, CA USA; 2 Department of Psychology, University of Sydney, Camperdown, New South Wales Australia; 3 Department of Psychiatry, Robert Wood Johnson Medical School, Rutgers, The State University of New Jersey, Piscataway, NJ USA; 4 Brain Health Institute, Rutgers Biomedical Health Sciences, Rutgers, The State University of New Jersey, Piscataway, NJ USA; 5 Department of Psychology, Rutgers, The State University of New Jersey, Piscataway, NJ USA

**Keywords:** Reward, Motivation, Neural circuits

## Abstract

Dopamine neurons in the ventral tegmental area support intracranial self-stimulation (ICSS), yet the cognitive representations underlying this phenomenon remain unclear. Here, 20-Hz stimulation of dopamine neurons, which approximates a physiologically relevant prediction error, was not sufficient to support ICSS beyond a continuously reinforced schedule and did not endow cues with a general or specific value. However, 50-Hz stimulation of dopamine neurons was sufficient to drive robust ICSS and was represented as a specific reward to motivate behavior. The frequency dependence of this effect is due to the rate (not the number) of action potentials produced by dopamine neurons, which differently modulates dopamine release downstream.

## Abstract (web summary)

Physiologically relevant stimulation of dopamine neurons does not function as a reward and does not endow cues with a reward representation. However, high-frequency stimulation is represented as a sensory-specific goal that motivates behavior.

## Main

During learning, dopamine neurons in the ventral tegmental area (VTA_DA_ neurons) exhibit a phasic prediction-error signal when something unexpected occurs and we need to learn about it. This signal exhibited by VTA_DA_ neurons is very brief and typically represents a firing rate of between 10 and 20 Hz (refs. ^1,2^). Traditionally, this signal has been studied in the context of reward learning, in which it is viewed as a representation of the scalar value in the unexpected reward, which allows cues preceding the reward to become valuable and more likely to be approached in the future. However, recent studies have revealed that this signal can function to support more general learning phenomena, including the development of cognitive maps^3^^,^^4^. Indeed, the dopamine prediction error appears to be able to support learning without making cues valuable at all^4^. However, if the dopamine prediction-error signal is not a value signal, why does dopamine appear valuable in some settings? For example, why is it that rats will work to receive phasic stimulation of VTA_DA_ neurons (that is, intracranial self-stimulation (ICSS))^5,6^, which suggests that this signal has value?

Surprisingly, although many studies have demonstrated ICSS with VTA_DA_ neurons, very few studies have investigated how this stimulation is represented in the brain. To test the nature of the representation supporting ICSS of VTA_DA_ neurons, we investigated how dopamine stimulation would interact with the well-studied Pavlovian-to-instrumental transfer (PIT) effect, which is seen in both rodents and humans and facilitates differentiation between specific and general representations of rewards^7,8^. The PIT procedure involves first teaching subjects that two cues lead to different types of rewards (for example, grain pellets or sucrose). Then, subjects separately learn that they can earn these two rewards by performing two different actions (for example, a left or right lever press). Finally, subjects are given a test in which the cues are played and two actions are available but do not produce their associated reward. This test allows us to probe the nature of the reward representation that contributed to learning in the earlier phases of the task. Specifically, if the reward was capable of driving learning by evoking a sensory-specific representation of itself, presenting the cue should motivate the subject to perform the action associated with the same reward. However, if the learning was devoid of a specific representation of reward identity and driven solely by a more general value mechanism, cue presentation would motivate lever-press responding in a nonspecific way, increasing responding on both levers.

Armed with this knowledge, we redesigned this selective PIT test in rats to include VTA_DA_ neuron stimulation as one of the rewards and compared this to a food reward. This would allow us to interrogate the cognitive representation that underlies ICSS of VTA_DA_ neurons. Against this backdrop, we stimulated VTA_DA_ neurons with two stimulus frequencies. One group received a 1-s 20-Hz stimulus train to VTA_DA_ neurons, which approximates a prediction error seen during learning about palatable rewards^2,4,9^ (although it is rather longer, as prediction-error signals are usually approximately 300–600 ms)^2^. A second group received a 1-s 50-Hz stimulation train to dopamine neurons, which is a supraphysiological rate typically used to show ICSS^5,6,10,11^. In fact, studies often use frequencies even above this supraphysiological range^5,11^. This allowed us to test whether dopamine stimulation is represented as a specific reward within the brain, how it compares to a natural food reward, and whether this differs between a physiological and a supraphysiological frequency.

**Fig. 1:**
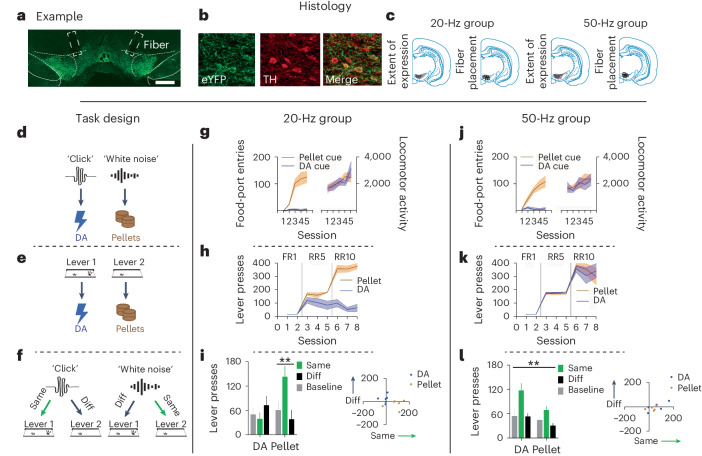
Stimulation of dopamine neurons at 20 Hz is not sufficient to promote ICSS beyond a continually reinforced schedule and does not support PIT; however, a supraphysiological frequency of 50 Hz supports robust ICSS and is encoded as a specific sensory event. **a**–**c**, Histological verification of bilateral Cre-dependent ChR2 expression in VTA_DA_ neurons (scale bar, 1 mm); example of bilateral virus expression and fiber placement in VTA (**a**); colocalization of TH and viral expression (enhanced yellow fluorescent protein; eYFP), which approached ~90% (**b**); and a schematic of the minimal and maximum viral expression and fiber placement across rats (**c**). **d**–**f**, Task design using one counterbalancing example, which consisted of Pavlovian conditioning (**d**), instrumental conditioning (**e**) and the PIT test (**f**). Rats first learned that two auditory cues (for example, click and white noise) lead to two outcomes (for example, dopamine (DA) stimulation and pellets, respectively). Then, they learned to perform two lever presses that led to the two outcomes. Finally, rats were presented with the two auditory cues and given an opportunity to press either lever without reward feedback. **g**–**i**, Results for the 20-Hz group. Rats in the 20-Hz group (*n* = 6) increased food-port entries during the pellet-paired cue. These rats showed equivalent increases in locomotor activity across learning for both cues (**g**). During instrumental conditioning, rats in the 20-Hz group showed robust lever presses for pellets but not for dopamine stimulation (**h**). In the PIT test, when the pellet-paired cue was presented, these rats showed increased responding on the pellet-paired lever, indicating specific PIT. However, they did not show PIT for the dopamine-paired cue. Individual data points are represented in the scatterplot to the right of the bar graph (**i**). **j**–**l**, Results for the 50-Hz group. Rats in the 50-Hz group (*n* = 5) showed increases in food-port entries during the pellet-paired cue but not during the dopamine-paired cue. Increases in locomotor activity across learning were similar for the dopamine- and pellet-paired cues (**j**). During instrumental training, the 50-Hz group showed robust lever pressing for both dopamine stimulation and pellets (**k**). In the PIT test, the dopamine- and pellet-paired cues both produced robust specific PIT. Individual data points are represented in the scatterplot to the right of the bar graph (**l**). Data in **g**–**i** were replicated between subjects in Supplementary Fig. 2. Data were analyzed with a repeated-measures ANOVA and follow-up simple-effect analyses, where appropriate. **P < 0.05. Error bars indicate the s.e.m.

Before training, we infused 2 μl of a Cre-dependent adeno-associated virus (AAV) carrying the excitatory opsin channelrhodopsin-2 (ChR2; pAAV5-Ef1a-DIO-hChR2(E123T/T159C)-eYFP) bilaterally into the VTA of male and female rats expressing Cre recombinase under the control of the tyrosine hydroxylase (TH) promoter^4,9^ (*n* = 11; Fig. 1a–c; for power analyses, see Methods and Supplementary Fig. 1). During this surgery, we also implanted optic fibers bilaterally into the VTA. This allowed us to stimulate VTA_DA_ neurons through blue-light delivery (473 nm, 16 mW, 1 s). Rats first learned that the two auditory cues lead to either sucrose pellets or VTA_DA_ stimulation (counterbalanced). We recorded locomotor activity and the number of food-port entries (Fig. 1g,j). Locomotor activity increased across sessions for both cues (main effect, session: *F*_4,36_ = 8.595, *P* < 0.0001), with no difference in the degree of locomotor activity evoked by the cues (main effect, dopamine versus food: *F*_1,9_ = 0.075, *P* = 0.790) or any between-group difference in locomotor activity (main effect, 20 versus 50 Hz: *F*_1,9_ = 0.482, *P* = 0.505; Fig. 1g,j). The lack of a difference in locomotor activity between the 20- and 50-Hz conditions might suggest that phasic (as opposed to tonic^12^) dopamine does not determine the motivational vigor assigned to cues. Entries into the food port were seen only for the food-paired cue and not the dopamine-paired cue. A repeated-measures analysis of variance (ANOVA) of the data on food-port entries showed a significant main effect of cue (dopamine versus food: *F*_1,9_ = 13.221, *P* = 0.005), with no interaction by group (*F*_1,9_ = 0.105, *P* = 0.753). Similarly, there was a main effect of session (*F*_4,36_ = 10.425, *P* = 0.000) and a session by cue interaction (*F*_4,36_ = 9.196, *P* = 0.000) with no interactions by group (session × group: *F*_4,36_ = 0.522, *P* = 0.720; session × cue × group: *F*_4,36_ = 0.262, *P* = 0.900). Finally, there was no between-group difference in the overall responding in the food port (*F*_1,9_ = 0.106, *P* = 0.752). This shows that locomotor activity increased across learning for both cues in both groups, and only the food-paired cue promoted entries into the food port.

After this training, rats moved on to the instrumental contingencies, in which they could press one lever to receive dopamine stimulation and another lever to receive sucrose pellets. One lever was presented at a time, and rats could earn a maximum of 40 rewards, in line with other PIT studies^13^. This also helped us prevent competition between the two instrumental actions. The effort required to earn the rewards increased across training, from one lever press (fixed ratio 1 (FR1)) to an average of five (random ratio 5 (RR5)) and then ten (RR10) lever presses. This progressive schedule is typically used to establish a robust baseline of performance that will withstand the PIT test that takes place without reward deliveries^13^. These parameters render the rats sensitive to the number of lever presses needed to retrieve the same reward (that is, sensitive to increased effort) and that is dissociable from temporal delays^14,15^. During instrumental training, significant differences between our 20- and 50-Hz groups emerged (Fig. 1h,k). While rats in the 20-Hz group reached the criterion on FR1 schedules, similar to other reports^16–18^, their performance was not robust on leaner schedules. This was despite their maintenance of responding on these schedules for the food reward. However, rats in the 50-Hz group continued to show robust performance for both rewards as instrumental training progressed. This was confirmed with statistical analyses, in which a repeated-measures ANOVA demonstrated a significant main effect of reward (dopamine versus food: *F*_1,9_ = 21.528, *P* = 0.001) and a reward × group interaction (*F*_1,9_ = 21.544, *P* = 0.001) due to a significant difference in responding for dopamine stimulation between the 20- and 50-Hz groups (*F*_1,9_ = 30.738, *P* = 0.000), which was not present when comparing responding for food rewards (*F*_1,9_ = 0.128, *P* = 0.729). This same pattern was also seen when considering the increase in responding across sessions, which returned a main effect of session (*F*_7,63_ = 149.418, *P* = 0.000) and a session × group interaction (*F*_7,63_ = 9.413, *P* = 0.000) due to a difference in responding emerging between groups as instrumental training progressed (for example, session 8: *F*_1,9_ = 7.629, *P* = 0.022). There was also a between-group difference in the overall levels of responding (*F*_1,9_ = 15.647, *P* = 0.003). Thus, while 50-Hz dopamine stimulation functioned to motivate vigorous responding that was comparable to food rewards, consistent with other reports^5,10,11,19,20^, 20-Hz dopamine stimulation did not support robust instrumental responding beyond a continuously reinforced schedule and was not comparable to a food reward. While other studies have shown that rodents will nose poke for a 20-Hz stimulus train thousands of times across a session^17^^,^^18^^,^^21^, when the schedule is increased (as in the present study), rats will cease responding quickly^21^. Indeed, our same rats that would not increase their lever-press responding for the 20-Hz stimulation train showed a very high number of nose pokes, comparable to other studies^17^, on an FR1 schedule (Supplementary Fig. 5). Thus, increasing the reinforcement schedule reveals the reduced ‘value’ of the 20-Hz stimulation train.

Following training, rats received the PIT test, during which the dopamine- and food-paired cues were played, and rats had the opportunity to press either lever. During this test, neither the cues nor the levers produced their associated reward, which allowed us to investigate the nature of the reward representation driving behavior^7^. In our 20-Hz group, we found that the dopamine-paired cue did not produce specific PIT (Fig. 1i); rats did not increase their responding on either lever when the dopamine-paired cue was presented. However, when the food-paired cue was presented, these same rats selectively increased their responding on the lever that produced the food, demonstrating selective PIT. In contrast, in our 50-Hz group, we found that the dopamine-paired cue supported robust selective PIT, which was comparable to that produced by the food-paired cue (Fig. 1l). A repeated-measures ANOVA comparing responding in our 20-Hz group on the same and different levers, relative to the baseline, revealed a cue by lever interaction (cue × lever: *F*_1,5_ = 15.383, *P* = 0.011) due to a selective increase on the same lever in response to the food-paired cue (same versus different: *F*_1,5_ = 13.957, *P* = 0.013), which was not present for the dopamine cue (*F*_1,5_ = 3.759, *P* = 0.110). Importantly, there was also a significant difference when comparing responding on the same lever for the dopamine-paired cue versus the food-paired cue (*F*_1,5_ = 7.885, *P* = 0.038), which was not seen for the different lever (*F*_1,5_ = 3.243, *P* = 0.132). This same analysis conducted on the responding exhibited by our 50-Hz group revealed a main effect of lever (same versus different: *F*_1,4_ = 20.193, *P* = 0.011) but no main effect of cue (dopamine versus food: *F*_1,4_ = 0.585, *P* = 0.487) or any interaction (*F*_1,4_ = 0.505, *P* = 0.516). Thus, stimulation of dopamine neurons at 20 Hz did not function as a specific reward that could support instrumental lever pressing in the same way that a natural reward could. Further, 20 Hz did not endow the cue with motivational significance that could support PIT. Importantly, we also replicated this effect in a new cohort of rats, which we trained with either the 20-Hz stimulation or food outcome (Supplementary Fig. 2), ruling out any confounds associated with the comparison of these rewards. Interestingly, stimulating dopamine neurons at 50 Hz, which is typically used for ICSS studies^5,11^, supported robust instrumental responding on leaner schedules and selective PIT. This demonstrated that stimulation of dopamine neurons at a supraphysiological frequency of 50 Hz during ICSS produces a supraphysiological sensory event that is capable of acting as a representation of a specific reward to motivate behavior, over and above any role for this signal in endowing antecedent cues with a general value. This finding (in rodents) does not reflect our subjective everyday experience during reinforcement learning.

**Fig. 2:**
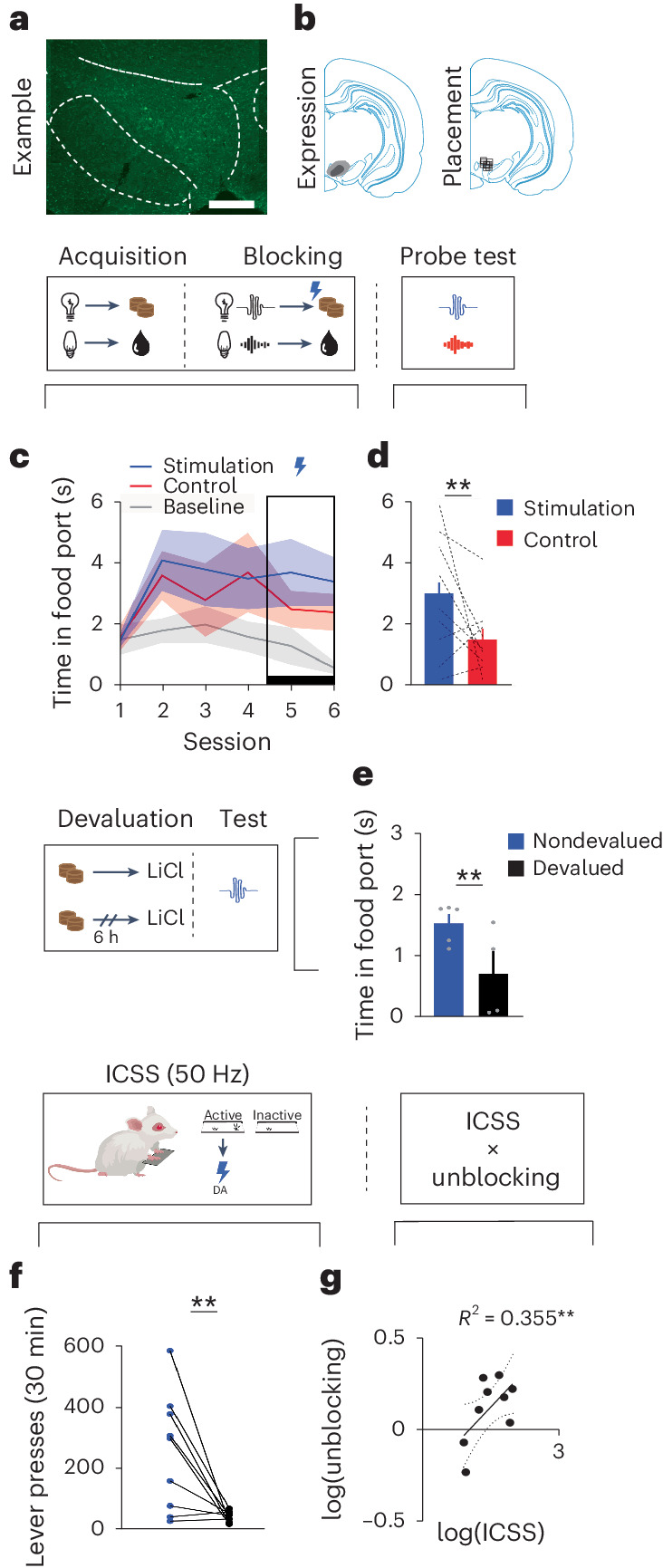
Stimulation of dopamine neurons at 20 Hz functions as a teaching signal to drive sensory-specific learning. **a**, Unilateral example of bilateral ChR2 expression in dopamine neurons (scale bar, 1 mm). **b**, Left, minimum and maximum viral expression across rats. Right, placement of fiber tips across rats. The schematic above **c** and **d** shows the design of our blocking task using one counterbalancing example, which consisted of acquisition, blocking and a probe test. **c**, Rats (*n* = 9) first learned that two visual cues lead to two distinct rewards (acquisition). Then, two new auditory stimuli were introduced in compound with the visual cues and led to the same rewards (blocking). During blocking, we stimulated dopamine neurons (1 s, 20 Hz) at reward delivery for one of the compounds (‘stimulation’ cue). Rats acquired the food-port response during the cues and maintained high levels of responding after the introduction of the auditory cues and dopamine stimulation. **d**, We next subjected rats to a probe test and found that they responded more to the stimulation cue relative to the control cue. The schematic to the left of **e** shows the devaluation procedure, which was used to devalue the reward paired with the stimulation cue using lithium chloride (LiCl). **e**, Rats showed a significant reduction in responding to the stimulation cue following devaluation, confirming that dopamine stimulation unblocked the association between the cue and a sensory-specific representation of the reward. The schematic above **f** and **g** shows the design used for ICSS. Rats were provided access to a lever that produced 50-Hz stimulation of dopamine neurons (active) or nothing (inactive). **f**, Rats pressed more on the active lever. **g**, This ICSS measure was positively correlated with the unblocking effect in (**d**) (Pearson’s correlation: *r* = 0.56, *P* = 0.045). This experiment was replicated in Supplementary Fig. 4 with the rats used for the PIT experiment in Fig. 1. Data across sessions were analyzed with a repeated-measures ANOVA and follow-up simple-effect analyses, where appropriate. A one-tailed *t* test was used to determine the significance between two means with directional hypotheses. **P < 0.05. Error bars indicate the s.e.m. Scale bar, 1 mm.

Next, we wanted to confirm that our 20-Hz stimulation of dopamine neurons was effective. To do this, we ran an additional positive control experiment with a new cohort of rats to test whether our 20-Hz stimulation of dopamine neurons can act as a teaching signal to produce learning, as we and others have previously demonstrated^3,4,9,16^. Here, we used the blocking procedure^22^. Rats first learned that two light cues lead to two different rewards (Fig. 2, ‘acquisition’; for example, house light → sucrose pellet, flash → grain pellet; counterbalanced). Then, we presented the lights in compound with new auditory cues, which produced the same rewards (for example, Fig. 2, ‘blocking’; house light + white noise → sucrose pellet, flash + click → grain pellets; counterbalanced). Usually, rats do not learn about the new auditory cues, as they are redundant. However, we stimulated dopamine neurons (1 s, 20 Hz) as one of the rewards was delivered. This created a ‘stimulation cue’, which we could compare to our ‘control cue’ that was not paired with stimulation of dopamine neurons. All rats learned to enter the port while the visual cues were presented, which was unaffected by the introduction of the auditory cues or dopamine stimulation (Fig. 2c; time period: *F*_2,16_ = 11.072, *P* = 0.001; session: *F*_5,40_ = 1.389, *P* = 0.249; time period × session: *F*_10,80_ = 2.710, *P* = 0.006; stimulation versus baseline: *P* = 0.006, control versus baseline: *P* = 0.003, stimulation versus control: *P* = 0.140). This demonstrated that all rats learned that the visual cues were predictive of reward and that the stimulation of dopamine neurons did not interfere with the rats’ ability to continue to respond to the food-predictive cues.

We then tested responding to the auditory cues alone without reward to assess how much learning had accrued toward them (Fig. 2, ‘probe test’). Here, we saw that rats spent more time in the food port during the stimulation cue relative to the control cue (Fig. 2d; *F*_1,8_ = 3.614, *P* = 0.047). This showed that stimulation of dopamine neurons was capable of driving additional learning to the stimulation-paired cue. To further probe the nature of the association that had developed through dopamine stimulation, we devalued the reward paired with the unblocked cue (Fig. 2, ‘devaluation’)^9^. Then, we presented the unblocked cue and found that rats in the devalued group spent less time in the food port during the cue (Fig. 2e; group: *F*_2,__7_ = 5.15, *P* = 0.029), suggesting that the cue responding was mediated by a representation of the reward. This was not seen when we tested responding to the blocked control cue, whose associated reward had not been devalued, confirming the sensory-specific nature of the effect (Supplementary Fig. 3, left; group: *F*_2,7_ = 0.157, *P* = 0.352). Together, these data show that our physiologically relevant stimulation could act as a teaching signal to drive sensory-specific associations between events despite not being able to function as a reward in itself.

In these same rats, we wanted to determine whether the rewarding effects of high-frequency stimulation are related to the ability of these neurons to act as a physiological teaching signal. To test this, we examined how much the rats would press to obtain optogenetic stimulation of VTA_DA_ neurons at 50 Hz (Fig. 2, ‘ICSS’). We found that rats would press consistently for 50 Hz of dopamine stimulation (Fig. 2f; *F*_1,8_ = 10.42, *P* = 0.006). We hypothesized that the degree of lever presses for 50-Hz stimulation would be positively correlated with the unblocking effect (Fig. 2d). Indeed, when we compared the magnitude of the unblocking to the degree to which high-frequency dopamine stimulation would support ICSS, we found that these factors were strongly positively correlated (Fig. 2g; Pearson’s *r* = 0.596, *R*^2^ = 0.355; *P* = 0.045). We did not see this same relationship with ICSS and the blocked cue (Supplementary Fig. 3, right; Pearson’s *r* = 0.148, *R*^2^ = 0.022; *P* = 0.352). These data suggest that the differential effects of the 20- and 50-Hz stimulation parameters in Fig. 1 are due to the change in the frequency of firing of these neurons and not due to these different frequencies tapping into different populations of dopamine neurons.

**Fig. 3:**
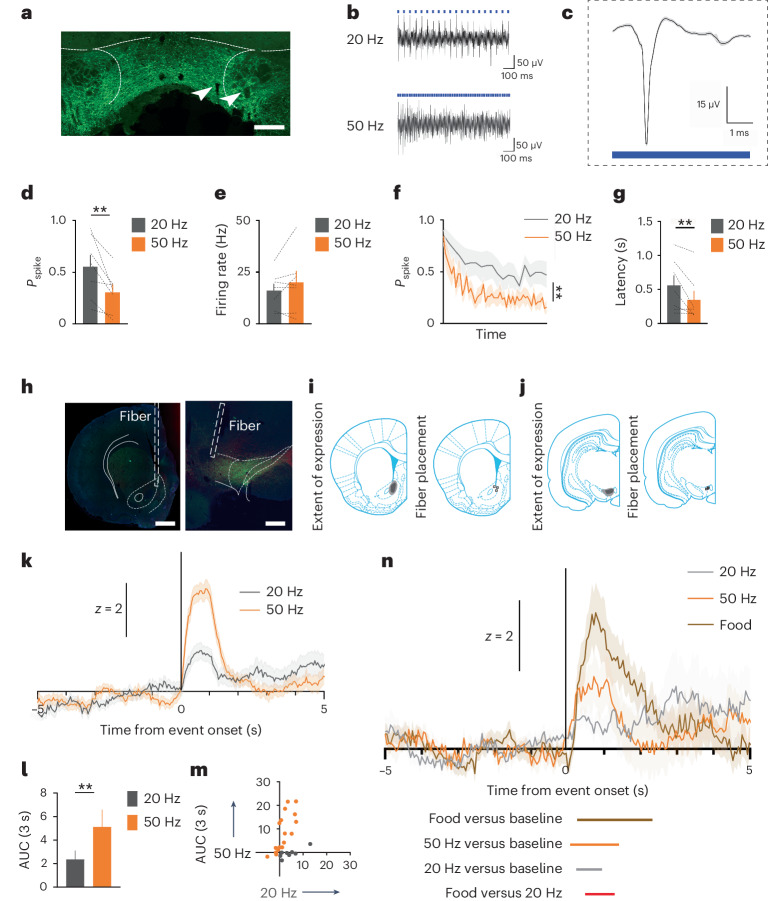
Optogenetic stimulation of VTA_DA_ neurons at 20 and 50 Hz results in the same number of action potentials across different timescales and produces differential dopamine release in the NAc. **a**–**g**, Simultaneous recording and stimulation of VTA_DA_ neurons (*n* = 7 cells). **a**, Example of bilateral Cre-dependent AAV-ChR2-eYFP expression (green) and microwire placement (indicated by white arrows; scale bar, 1 mm). **b**, Example traces of the 20- and 50-Hz stimulation trains. **c**, Triphasic dopamine extracellular spike. **d**, Neuronal activity follows the 20-Hz stimulus train more faithfully than the 50-Hz train, evidenced by the higher probability of a spike following each light pulse in the 20-Hz stimulus train. **e**, There was no difference in the overall number of action potentials generated by the 20- and 50-Hz stimulus trains. **f**, The rate of neuronal firing in VTA_DA_ neurons decreased more rapidly across the 50-Hz stimulation train relative to the 20-Hz train. **g**, The 50-Hz stimulation train resulted in greater burst firing, evidenced by a reduced latency to evoke the first ten action potentials. **h**–**n**, Simultaneous stimulation of VTA_DA_ neurons and recording of dopamine release in the NAc (*n* = 3 rats). **h**, Example of unilateral viral expression of ChR2 in VTA_DA_ neurons (left) and GRAB_DA_ in the NAc (right; scale bars, 1 mm). **i**, Histological verification of GRAB_DA_ expression and fiber placement in the NAc. **j**, Histological verification of ChR2 expression and fiber placement in the VTA. **k**, Temporal dynamics of dopamine release in the NAc as a consequence of 20- or 50-Hz stimulation of dopamine neurons. **l**, Quantification of AAV-hSyn-GRAB_DA2m_ fluorescence as the AUC of the *z*-scored data seen in the NAc after 20- and 50-Hz stimulation, which revealed greater dopamine release following the 50-Hz stimulus train. **m**, Individual data points for **l**. **n**, Temporal dynamics of dopamine release in the NAc in response to the 20-Hz train, the 50-Hz train and a food pellet. Waveform analyses revealed significance, as indicated by the colored bars below the traces. Periods of significance from baseline were defined by the bootstrapped 95% CIs^30^. The significance between events (that is, food versus 20 Hz) was determined by permutation tests. We did not replicate these data; however, **d**–**f** and **k**–**m** are similar to prior reports^29,31^. Unless otherwise noted (that is, in **n**), data were analyzed using repeated-measures ANOVA. **P < 0.05. Error bars indicate the s.e.m.

We then established how VTA_DA_ neurons respond to our differential stimulation parameters. We infused a Cre-dependent AAV carrying ChR2 into the VTA of TH-Cre rats. During this surgery, rats were implanted with a 16-channel microwire array with a central optic fiber into the VTA, which would afford simultaneous stimulation and recording of dopamine neurons (Fig. 3a–c). Similarly to prior results^20^, we found that the 20-Hz stimulation produced firing rates of greater fidelity to the stimulation parameters compared to the 50-Hz stimulation (Fig. 3d; *F*_1,6_ = 12.38, *P* = 0.013). Interestingly, the firing rates of the neurons did not significantly differ overall across the 1-s stimulus train in response to the 20- versus 50-Hz stimulation trains (Fig. 3e). While there was no difference in the spike probability within the first five light pulses (Fig. 3f; *F*_1,6_ = 4.7, *P* = 0.074), a significant difference emerged toward the end of the stimulus train in the last five pulses (Fig. 3f; *F*_1,6_ = 11.61, *P* = 0.014). Consistent with this observation, there was also a difference in the latency at which the stimulus trains produced the first ten action potentials. Specifically, the 50-Hz train resulted in a reduced latency to the first ten spikes relative to the 20-Hz train, reflecting greater burst-like firing early in the 50-Hz train (Fig. 3g; *F*_1,6_ = 7.983, *P* = 0.03). These data show that the different stimulation parameters produce a similar number of action potentials, but the 50-Hz stimulation parameters produce them across a shorter time period. This demonstrates that it is the higher frequency (and not the greater number of spikes) that generates the difference in the ability of these stimulation parameters to drive reinforcement.

Next, we examined how our 20- and 50-Hz stimulation parameters differed in terms of dopamine release downstream. We infused a Cre-dependent AAV carrying ChR2 into the VTA and the G-protein-coupled receptor-activation-based dopamine (GRAB_DA_) sensor (AAV9-hSyn-GRAB_DA2m_) into the medial core of the nucleus accumbens (NAc) (anterior–posterior (AP), 1.3; medial–lateral (ML), −1.3; dorsal–ventral (DV), −7.2 and −6.4) of TH-Cre rats (*n* = 3; Fig. 3h–j; post hoc analysis, power (1 − *β*) = 0.87). During this surgery, we also placed a fiber-optic implant into the VTA and NAc (AP, 1.3; ML, −1.3; DV, −6.8). Here, we found that 50-Hz stimulation of dopamine neurons resulted in a substantially higher release of dopamine relative to 20-Hz stimulation, consistent with other reports^23^ (Fig. 3k–m; area under the curve (AUC), *t* statistic, 20 versus 50 Hz: 2.47, *P* = 0.017). This showed that the faster rate of action potentials in VTA_DA_ cell bodies elicited by 50-Hz stimulation was associated with greater dopamine release downstream despite the overall number of action potentials being the same.

Finally, we investigated how dopamine release in the NAc, seen with our different stimulation parameters, would compare to food delivery. We performed waveform analyses on these traces that determine statistical significance through the bootstrapped 95% confidence intervals (CIs)^24^. We found that food delivery evoked a significant increase in dopamine for a prolonged period (Fig. 3n, brown bar below the traces, food versus baseline). The 50-Hz stimulation of VTA_DA_ neurons also evoked a significant increase in dopamine release but over a shorter time period than food delivery (Fig. 3n, orange bar, 50 Hz versus baseline). Similarly, the 20-Hz stimulation train produced a significant increase in dopamine release above baseline but, again, over a shorter timescale than both the 50-Hz train and food delivery (Fig. 3n, gray bar, 20 Hz versus baseline). Finally, permutation tests comparing the magnitude of dopamine release evoked by the events revealed a significant difference between food and the 20-Hz train (Fig. 3n, red bar, food versus 20 Hz) but not between food and the 50-Hz train. Thus, food delivery evoked a significant increase in dopamine release downstream, which was more prolonged than that evoked by either the 20- or 50-Hz stimulation train but differed in magnitude only from that elicited by the 20-Hz stimulation train. This suggests that the dopamine release in response to the 20-Hz stimulus train, which approximates a prediction error seen in VTA_DA_ neurons during receipt of food rewards^1^^,^^2^, does not capture the complexity and magnitude of dopamine release seen with food rewards themselves. This dissociates dopamine release resulting from the prediction-error teaching signal from that seen with unexpected food itself, which likely reflects additional modulation within the NAc itself^11^.

The present results are important for two reasons. First, they show that 20-Hz stimulation of VTA_DA_ neurons (approximating the error signal during reward receipt), as well as the corresponding dopamine release downstream, does not function as a reward comparable to food^25^, drugs^26^ or even higher frequencies of dopamine stimulation. Further, cues paired with either 20 or 50 Hz of dopamine stimulation did not invigorate indiscriminate responding during our PIT tests, showing that phasic dopamine signals do not endow cues with a general value. That is, the value hypothesis argues that the dopamine error signal reflects the magnitude of the reward’s scalar value^1^, which requires that firing in dopamine neurons functions as a reward in its own right in a way that can assign a scalar (general) value to an antecedent cue. Thus, even in the context of ICSS, in which the dopamine signal functions as an outcome, a learning-relevant 20-Hz signal^2,4,9^ does not (by itself) function as a reward that possesses sufficient reinforcing properties necessary to convey the value signal. Second, these results shed light on the psychological basis of ICSS using a 50-Hz stimulation train, which constitutes a high frequency of dopamine stimulation. These data demonstrate that using 50-Hz stimulation in the context of ICSS functions to create a sensory event that acts as a specific reward in its own right. This is consistent with data showing that dopamine release in response to high-frequency stimulation is regulated by error mechanisms in the same way as other rewards^10,27^. Here, we additionally reveal the cognitive representation that drives this effect. This finding does not have a basis in our everyday learning experience. Put simply, our learning experience does not contain representations of phasic dopamine as a rewarding sensory event. This demonstrates that the study of high-frequency stimulation of VTA_DA_ neurons does not inform us of the role of prediction errors in reinforcement learning. More generally, it begs the following question: is there any physiological experience that might relate to ICSS? A circumstance in which this may perhaps become relevant is drug seeking^19,28^, where most drugs of abuse act, in at least some way, to increase phasic dopamine activity^29^. If so, these data support the idea that people with substance use disorder seek out drugs of abuse to obtain a specific sensory experience, not because the actions or cues associated with the drug have become valuable.

## Methods

### Subjects

Forty experimentally naive male and female Long–Evans rats sourced from Charles River Laboratories were used for the experiments. Of these, 33 were transgenic rats carrying a TH-dependent Cre-expressing system^4^^,^^17^, which were originally sourced from the Rat Resource and Research Center before breeding at the University of California, Los Angeles (UCLA). Rats were approximately 4 months of age before the surgical procedures. The animals were maintained on a 12-h light–dark cycle, in which all behavioral experiments took place during the light cycle. Rats had ad libitum access to food and water unless undergoing the behavioral experiment, during which they received sufficient chow to maintain them at ~85% of their free-feeding body weight. Rats were randomly assigned to groups, and we ensured that groups were sex- and age-matched. All experimental procedures were conducted in accordance with the guidelines of the UCLA Animal Research Council and/or the Rutgers University Animal Care and Use Committee.

### Surgical procedures

Surgical procedures have been described elsewhere^4,32^. Briefly, rats were anesthetized with isoflurane (3% induction, 1–2% maintenance in 1 l min^−1^ O_2_) and secured in a stereotaxic device (David Kopf Instruments). Rats received two infusions of 1.0 µl (bilaterally, 2.0 µl per hemisphere) AAV5-EF1α-DiO-ChR2-eYFP (E123T/T159C) (*n* = 33; item ID 26966-AAV5, titer 2.7 × 10^12^) into the VTA at the following coordinates: AP, −5.3 mm; ML, ±0.7 mm; DV, −6.5 and −7.7 mm (females) or −7.0 and −8.2 mm (males). The virus was obtained from Addgene. For the behavioral experiments involving PIT and blocking, optic fibers (Thorlabs, 200-µm diameter) were implanted bilaterally at the following coordinates relative to the bregma: AP, −5.3 mm; ML, ±2.61 mm; DV, −7.05 mm (female) or −7.55 mm (male), at an angle of 15° pointed toward the midline. For the electrophysiological recording experiment, a 16-channel microwire array with a central optic fiber was implanted targeting the VTA (*n* = 4; −5 to −6.5 AP, +1.58 to 2.58 ML at a 10° angle, −7.9 DV; Microprobes), according to our previously published protocol^33^. For the recording of dopamine release in the NAc with fiber photometry during optogenetic stimulation of VTA_DA_ neurons, we also infused 0.5 µl of an AAV carrying GRAB_DA_ (*n* = 3; AAV9-hSyn-GRAB_DA2m_, item ID 140553-AAV9, titer 1 × 10^13^) into the NAc in the same hemisphere (AP, +1.3; ML, +1.3; DV, −7.2 and −6.4) and placed a fiber-optic implant targeting the NAc medial core (AP, +1.3; ML, +1.3; DV, −6.8).

### Apparatus

Training was conducted in eight standard behavioral chambers, which were individually housed in light- and sound-attenuating boxes (Med Associates). For behavioral experiments, the chambers were equipped with a pellet dispenser that delivered 45-mg sucrose pellets (5TUT, BioServ) into a recessed magazine when activated. Two retractable levers could be inserted into the chambers on either side of the recessed magazine. Access to the magazine was detected by means of infrared detectors mounted across the opening of the recess. A computer equipped with MedPC software (Med Associates) controlled the equipment and recorded the responses. Raw data were outputted and processed in MPC2XL (Med Associates) to extract relevant response measures. The chambers contained a speaker connected to a white-noise generator and a relay that delivered a 5-kHz clicker stimulus, as well as a house light that could illuminate the chambers when programmed.

### Behavioral procedures

#### Pavlovian-to-instrumental transfer

The parameters for the PIT experiment was based on a previously described procedure^13,34^. Briefly, rats began training with Pavlovian conditioning, which continued for 10 days. During the 60-min session, rats received four 2-min presentations of two cues (clicker or white noise, counterbalanced), each paired with one of two rewards (dopamine simulation or sucrose pellets, counterbalanced). Rewards were randomly delivered at four points throughout the 2-min cue. To stimulate dopamine neurons, we delivered light (473 nm, 1 s, 14–16 mW) into the rats’ brains at either 20 or 50 Hz (5-ms pulses)^4,9^. Intertrial intervals (ITIs) averaged 5 min in duration. Locomotor activity was recorded throughout the session and analyzed using ezTrack^35^. Following Pavlovian conditioning, rats received 8 days of instrumental training, in which they were trained to lever press for pellets or dopamine stimulation. Here, rewards were delivered immediately after the lever press. As the rats could hear the audible turn of the pellet dispenser, the delay between food and dopamine rewards from the lever press should be the same. Each session consisted of two 10-min blocks on each lever, separated by a 2.5-min time-out period during which the levers were retracted. If an animal pressed a lever more than 20 times during a 10-min session, the lever was retracted immediately and the 2.5-min time-out period began before the next lever was made available, earning a maximum of 40 rewards on each lever. For the first 2 days of instrumental training, lever presses were continually reinforced. Rats were then moved on to 3 days of a random ratio schedule, in which each lever delivered a reward with a probability of 0.2 (that is, RR5) and, finally, to an RR10 schedule, which delivered a reward with a probability of 0.1. During the critical PIT test, both levers were extended, and no rewards were delivered. To extinguish instrumental responding on both levers, rats first received 8 min of extinction before cue presentation. Then, each cue was presented four times in the following order: clicker–noise–noise–clicker–noise–clicker–clicker, with a fixed 3-min ITI between cues. Rats received three such sessions, in which they received one RR10 session in between these tests.

#### Blocking

Rats first received a presentation of two light cues followed by the delivery of two distinct rewards (for example, flash → sucrose, house light → pellet; counterbalanced). Rats received eight sessions consisting of 14 trials, separated by a variable ITI of 4 min. Then, rats received the visual cues in compound with two new auditory cues to create two new audiovisual compounds, which were followed by the same rewards (for example, flash + click → sucrose, house light + white noise → pellets; counterbalanced). During compound training, rats received stimulation of dopamine neurons that coincided with reward delivery after one of the compounds^3,16^ (473 nm, 1 s, 20 Hz, 5-ms pulses, 14–16 mW) to mimic an endogenous prediction error^1,2,31^. Rats then received two probe tests with the click and white noise alone without reward, consisting of eight trials with an ITI averaging approximately 4 min. All stimuli were counterbalanced, as was the order of their presentation.

#### Devaluation

Following blocking, we trained rats on instrumental contingencies for the two distinct rewards. We then tested whether the cues would promote PIT. However, we did not see significant PIT in any direction, primarily because the rats spent a lot of time in the food port during the unblocked cue (blocked (±s.e.m.), 1.5 s (0.2 s); unblocked (±s.e.m.), 2.7 s (0.2 s); *F*_1,8_ = 6.230, *P* = 0.02). Instead, we conducted a devaluation test to assess the nature of the association that had developed between the unblocked cue and the reward. To do this, we allowed rats to consume the reward paired with the unblocked cue (paired with stimulation) in a new context for 30 min. Immediately thereafter, half the rats received injections of lithium chloride (0.15 M, 10 mg ml^−1^; devalued group). The remaining rats were returned to the colony room and received injections 6 h later (nondevalued group). We repeated this procedure for 3 days. After a 48-h recovery period, rats were again placed in the experimental chambers and given a final probe test, in which the unblocked cue was presented without reward. We also tested responding to the blocked cue (not paired with stimulation; Supplementary Fig. 3). Each cue was presented for a total of four trials, with an ITI averaging approximately 4 min.

#### Intracranial self-stimulation

To examine whether the rats from our blocking experiment would perform ICSS for 50-Hz stimulation of dopamine neurons, we allowed them access to a lever that would produce 1 s of 50-Hz stimulation (473 nm, 1 s, 14–16 mW, 5-ms pulse) on a continually reinforced schedule and another lever that would produce nothing. This session lasted for 30 min, and rats could press the levers as much as they wanted.

### Electrophysiological procedures

Traditional photo-tagging procedures have accounted for differences in the amount of light reaching proximal and distal microwires by titrating the light delivery for each wire^30,36^. However, we opted against this approach. Instead, we fixed the light intensity at 15 mW to provide an accurate model of neuronal firing in the tissue surrounding the optic fiber for the behavioral tasks used in the present study. Accordingly, we observed a decrease in fidelity for neurons distal to the optic fiber, consistent with models of optogenetic stimulation. Neurons were classified as ChR2-responsive if they reliably exhibited responses within 15 ms of the light onset^36^. To confirm the absence of photoelectric artifacts, we recorded two additional rats with wires implanted dorsal to the VTA. In these rats, we found no evidence of optically evoked activity, even at optical power levels above those used in the experimental preparation.

On the day of testing, rats were placed in a standard operant chamber and allowed to habituate for 10–15 min, during which neurons were identified and sorted online using Synapse software (Tucker-Davis Technologies (TDT)). Single neurons were amplified and recorded using a digital headstage (ZD32, TDT), a preamplifier (PZ5) and a bioamplifier (RZ2). Signals were then bandpass filtered at 300–5,000 Hz and stored using Synapse (TDT) at 24 kHz. Rats were first given ten trials of 20-Hz stimulation (473-nm laser, 15 mW, 5-ms pulse) with a 1-min ITI, followed by ten trials of 50-Hz stimulation (473-nm laser, 15 mW, 5-ms pulse) with a 1-min ITI.

### Fiber photometry procedures

We used a commercially available fiber photometry system (Neurophotometrics). The 470-nm excitation light was adjusted to approximately 80–100 µW at the tip of the patch cord (fiber core diameter, 200 µm; Doric Lenses). Fluorescence emission was passed through a 535-nm bandpass filter and focused onto the complementary metal-oxide semiconductor camera sensor through a tube lens. Samples were collected at 20 Hz using a custom Bonsai^37^ workflow. Time stamps of task events were collected simultaneously through an additional synchronized camera aimed at the Med Associates interface, which sent light pulses coincident with task events. Signals were saved using Bonsai software and exported to MATLAB (MathWorks) for analysis. During the recording sessions, rats were hooked up to our optogenetic lasers and fiber photometry systems. Rats received three ~40-min recording sessions, during which we measured dopamine release in the NAc medial core. Two of these sessions comprised the delivery of 20- and 50-Hz stimulation trains for 1 s into the VTA, whereas the third session involved the delivery of 20- and 50-Hz trains for 1 s, as well as the delivery of one food pellet simultaneously. Rats received 4–10 trials of each event, with a variable ITI approaching 2 min. Trial order was pseudorandomly counterbalanced such that no particular event could occur more than twice in a row. Rats that did not show sufficient signal variation in the 470-nm channel were discontinued from the experiment and excluded from all analyses.

### Histology

#### Behavioral and fiber photometry experiments

Rats were culled using carbon dioxide asphyxiation and perfused with phosphate buffer followed by 4% paraformaldehyde in phosphate buffer. Coronal sections (20 µm) were collected using a cryostat (Leica Biosystems), imaged and visualized for confirmation of viral expression and fiber-tip placements using a Zeiss LSM 900 microscope with a 4× or 20× objective and ZEN imaging software. Rats without sufficient viral expression and/or fiber placement were removed from all analyses.

#### Electrophysiological experiments

Rats were terminally anesthetized, and anodal current (50 µA, 4 s) was passed through each microwire to mark the tip location. Rats were then perfused with phosphate buffer (0.1 M, pH 7.4) followed by 4% paraformaldehyde. The brain was then stored in 4% paraformaldehyde overnight before storing in 18% sucrose at 4 °C until the brain equilibrated with the specific gravity of the sucrose solution. The VTA was then coronally sectioned at 30 µm to validate ChR2 expression and localize each microwire, as described previously^33^. Briefly, the positions of the microwires were identified by their tracks in the brain and their lesions at the microwire tips.

#### Immunohistological staining

To verify viral expression targeting dopamine cells, we processed brain tissues obtained from experimental animals and stained them for TH. Coronal sections (30 µm) of the VTA and NAc were collected using a cryostat (Leica Biosystems) into well plates containing 0.1 M PBS. Sections were first blocked using a solution composed of 3% normal goat serum (Millipore Sigma) and 0.2% Triton X-100 (Thermo Fisher Scientific) in 1× PBS before being incubated with the primary antibody (rabbit anti-TH, 1:1,000 concentration; Sigma-Aldrich) for 48 h at 4 °C. After this first incubation, sections were then allowed to incubate with the secondary antibody (goat anti-rabbit IgG Alexa Fluor 594, 1:500 concentration; Thermo Fisher Scientific) for 2 h. Following the final incubation, slices were mounted onto microscope slides and coverslipped with ProLong Gold reagent with DAPI (Thermo Fisher Scientific) before being imaged.

### Statistical analyses

#### Behavioral and electrophysiological data

Statistical analyses were conducted using the SPSS 24 IBM statistics package. Analyses were conducted using a mixed-design repeated-measures ANOVA. All analyses of simple main effects were planned and orthogonal and, therefore, did not require correction for multiple comparisons. One-tailed *t* tests were used for results with an a priori directional hypothesis. Data distribution was assumed to be normal, but homoscedasticity was not formally tested. PIT test data were analyzed across all trials of all three sessions of the PIT tests, and the blocking data were analyzed across the first six trials of both probe tests, consistent with other published demonstrations^16,38^. The ICSS and blocking data were correlated using Pearson’s correlations on the log(*x* + 1)-transformed data from the first 5 min of the ICSS session and the ratio of responding to the blocked or unblocked cue relative to baseline (that is, *X* − baseline/*X* + baseline). For the recording studies, neuronal fidelity was calculated by examining the number of pulses within each trial in which an action potential was recorded within 15 ms of the light onset. Fidelity was then further examined as a function of (1) each trial as a whole, (2) the pulse number in each train, and (3) the first five and last five pulses in each trial. The overall firing frequency across the 1-s train, irrespective of fidelity to individual pulses, was also analyzed. Finally, we examined the latency to the tenth action potential exhibited by our 20- and 50-Hz trains. On trials in which we did not see a tenth action potential, regression analyses were used to approximate the expected latency to the tenth action potential. Data collection and analyses were not performed blind to the conditions of the experiments. Sample sizes were chosen based on similar prior experiments that have elicited significant results with a similar number of rats^39^. Further, we ran a formal post hoc power analysis on the data elicited from these experiments, using G*Power 3.0 to estimate the power we had achieved using our sample sizes^40^. Specifically, we used the average of the partial *ƞ*^2^ (∼0.8) from our analyses from the PIT test with the 20- and 50-Hz groups to calculate the power (1 − *β*) we had achieved. These analyses revealed an estimated power of 0.99 with the same sizes used in our study (Supplementary Fig. 1), with a type 1 error rate (*α*) below 0.05. We also conducted these same analyses for our fiber photometry experiments, which also demonstrated an achieved power of >0.8.

#### Fiber photometry data

To smooth out the potential effects of photobleaching and motion artifacts, we collected an isosbestic control signal (415 nm) and a dopamine-dependent signal^41^ (470 nm). As the isosbestic signal also undergoes decay across a session with photobleaching, we can use this to correct the dopamine-dependent signal for photobleaching. We first removed the first 60 s of the data (before the onset of the first event), as this consistently had high variability in the signal and would disrupt baseline correction. The control signal was fit to linear regression and scaled to the GRAB_DA_ signal. We then subtracted the scaled control signal from the GRAB_DA_ signal and normalized it to obtain the change in the fluorescence response (Δ*F*/*F*). Using this Δ*F*/*F*, we extracted the signal of each trial aligned to events of reward delivery, 20-Hz stimulation and 50-Hz stimulation. We then *z*-scored the Δ*F*/*F* for each trial using the mean and s.d. of the signal of the 5-s time window before events. We calculated the AUC for the *z* scores across 3 s from event onset (20 and 50 Hz). To statistically analyze the data, we fit the data to a linear mixed-effects model in MATLAB, which allowed us to compare the AUCs for the 20- and 50-Hz stimulation train events across trials while factoring in the rat from which the signal came (that is, AUC ~ event + (1|ratID)). Waveform analyses were performed based on the MATLAB code from a previous study^24^. We defined the transients as significantly different from baseline (that is, the *z* score is zero) when the bootstrapped 95% CIs did not include 0 for more than 0.2 s. To compare transients induced by food and 20- or 50-Hz stimuli, we performed permutation tests. To compute *P* values for each time point, we randomly shuffled the signal data 1,000 times. We defined the difference between events as significant when *P* values were <0.05 for at least 0.2 s.

### Inclusion and ethics

We worked to ensure sex balance in the selection of nonhuman subjects. One or more of the authors identify as having a disability. The author list is gender balanced, and we worked to achieve gender balance in our reference list.

### Reporting summary

Further information on research design is available in the Nature Portfolio Reporting Summary linked to this article.

## Online content

Any methods, additional references, Nature Portfolio reporting summaries, source data, extended data, supplementary information, acknowledgements, peer review information; details of author contributions and competing interests; and statements of data and code availability are available at 10.1038/s41593-024-01643-1.

### Supplementary information

Supplementary Information Supplementary Figs. 1–5.

Reporting Summary

## Data Availability

Data are available upon request to melissa.sharpe@sydney.edu.au.

## Code availability

We used code freely available at https://github.com/philjrdb/ERTsimulation for the waveform analyses. Other code for our fiber photometry pipelines is available upon request.
