# Supplementary material for: Cognitive representations of intracranial self-stimulation of midbrain dopamine neurons depend on stimulation frequency
Source: Nat Neurosci. 2024 May 13;27(7):1253–9. doi: 10.1038/s41593-024-01643-1 (PMC11239488; doi:10.1038/s41593-024-01643-1)
Supplement: Supplementary file 1 — Supplementary Figs. 1–5. [file 41593_2024_1643_MOESM1_ESM.pdf]

nature neuroscience

Brief Communication

https://doi.org/10.1038/s41593-024-01643-1

Cognitive representations of intracranial self-stimulation of midbrain dopamine neurons depend on stimulation frequency

In the format provided by the authors and unedited

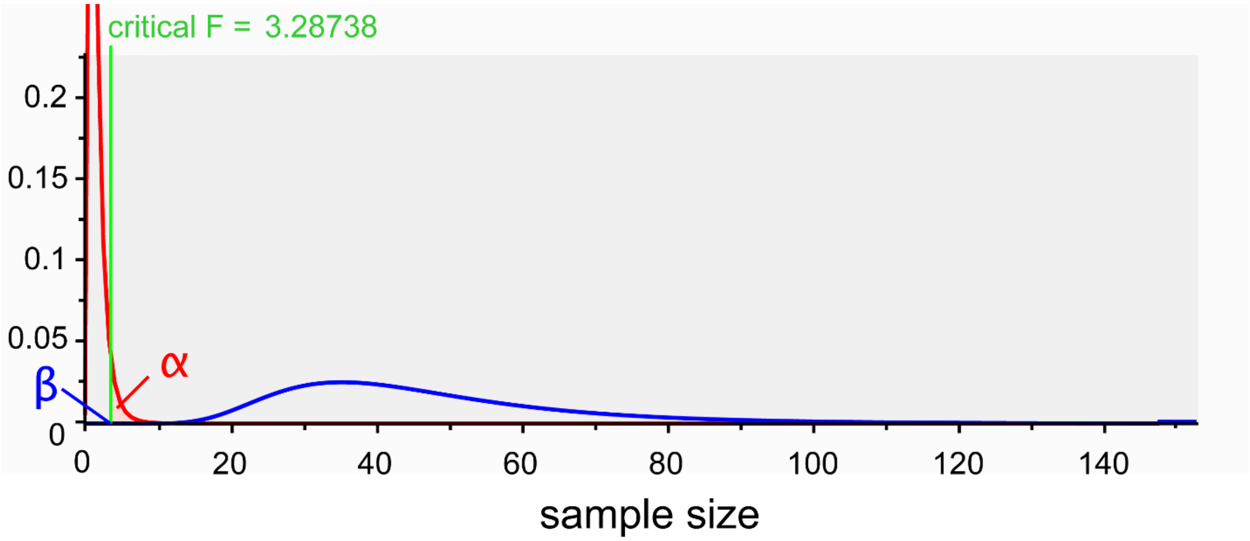

Fig. S1. Formal power analyses showed that we obtained a high degree of power with the sample sizes used in these studies. Using G*power 3.0[40], we conducted formal post-hoc power analyses on the data elicited from our PIT tests. The average partial η^2 elicited from our critical effects in our 20Hz and 50Hz groups was ~0.8, which resulted in a high degree of power (1-β; 0.99), and a type 1 error rate (α) below 0.05, with the sample sizes used in our study. This demonstrates that our sample sizes were sufficient to detect our critical effects with low likelihood of type 1 (α) or type 2 (β) errors.

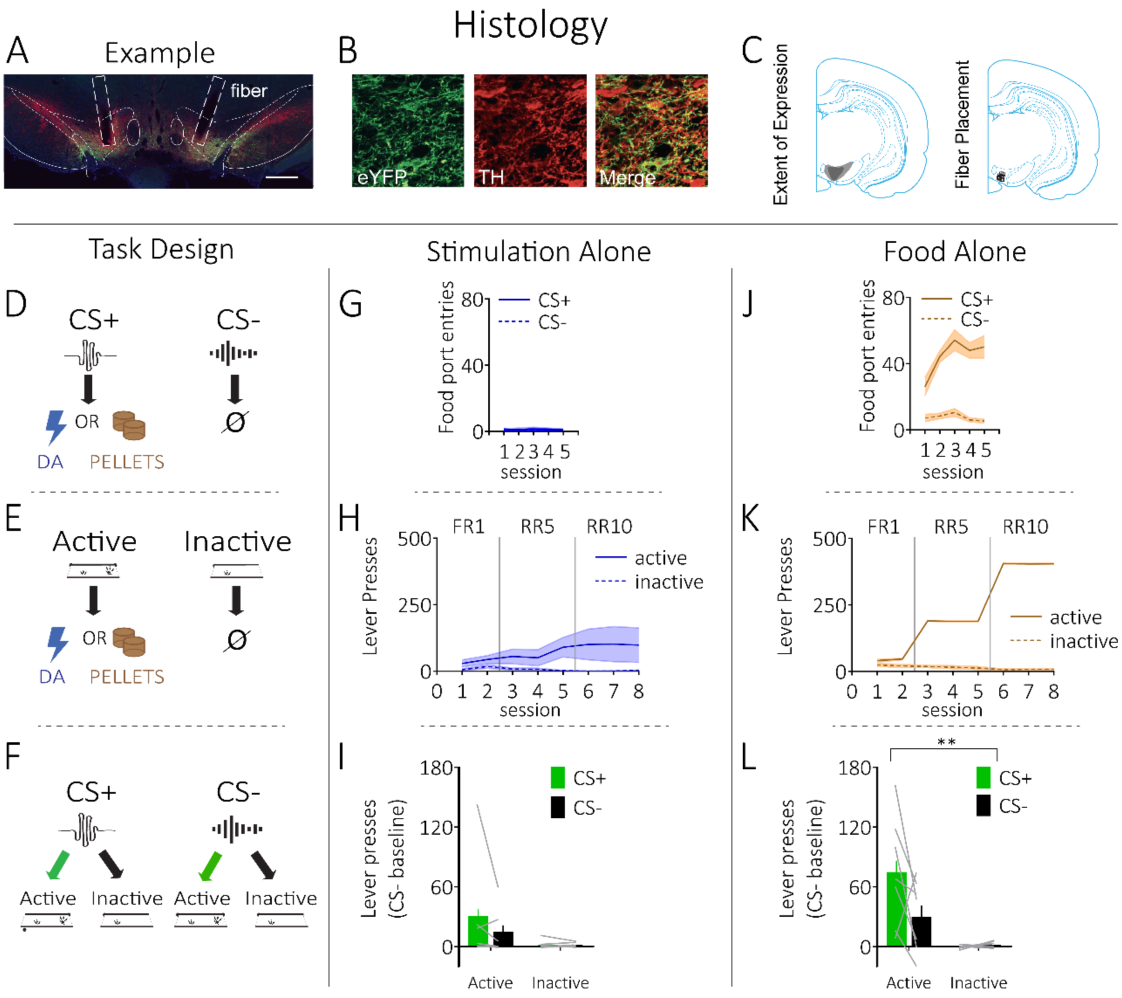

Fig S2. 20Hz stimulation of dopamine does not support robust ICSS and is not sufficient to produce PIT in a between-subjects procedure. Top: Histological verification. **A**) bilateral Cre-dependent ChR2 expression in TH-Cre rats, **B**) colocalization of TH and virus expression, and **C**) schematic of virus expression and fiber placement. **D)** Pavlovian conditioning. **E)** Rats in the stimulation-alone group (*n*=6) did not increase food-port entries, **F**) while the food-alone group did (*n*=7; repeated-measures ANOVA, CS: $F_{1,11}$=111.833, *p*=0.001; session: $F_{4,44}$=16.452, *p*=0.001; CS x group: $F_{1,11}$=104.055, *p*=0.001; group: $F_{1,11}$=176.241, *p*=0.001). **G**) Instrumental conditioning. **H**) Rats in the stimulation-alone group did not increase lever-press responding on the active lever for 20Hz stimulation as the reinforcement schedule progressed, **I**) which was in contrast to rats in the food-alone group (repeated measures ANOVA, lever: $F_{1,10}$=95.726, *p*=0.001; lever x group: $F_{1,10}$=28.025, p=0.001; lever x group x day: $F_{7,70}$=14.171, *p*=0.001; simple effect of day of active lever, food-alone: $F_{7,4}$=1076.891, *p*=0.001; simple effect of day x active lever, stimulation-alone: $F_{7,4}$=1.176, *p*=0.464. **J**) PIT test. **K**) rats in the stimulation-alone group did not show an elevation in responding on the active lever when the CS+ was presented, (Repeated measures ANOVA, CS: $F_{1,5}$=1.327, *p*=0.301; lever: $F_{1,5}$=1.715, *p*=0.247; CS x lever: $F_{1,5}$=1.255, *p*=0.314). **L**) However, rats in the food-alone group showed a selective increase on the active lever during the CS+, indicating PIT (Repeated measures ANOVA, CS x lever: $F_{1,6}$=4.504, *p*=0.039; simple effect, CS x active $F_{1,6}$=13.731, *p*=0.010; simple effect, CS x inactive: $F_{1,6}$=4.819, *p*=0.071). This experiment is a between-subjects replication of Fig 1G-I. Error bars =SEM. **indicates p<0.05. Scale bar = 1mm.

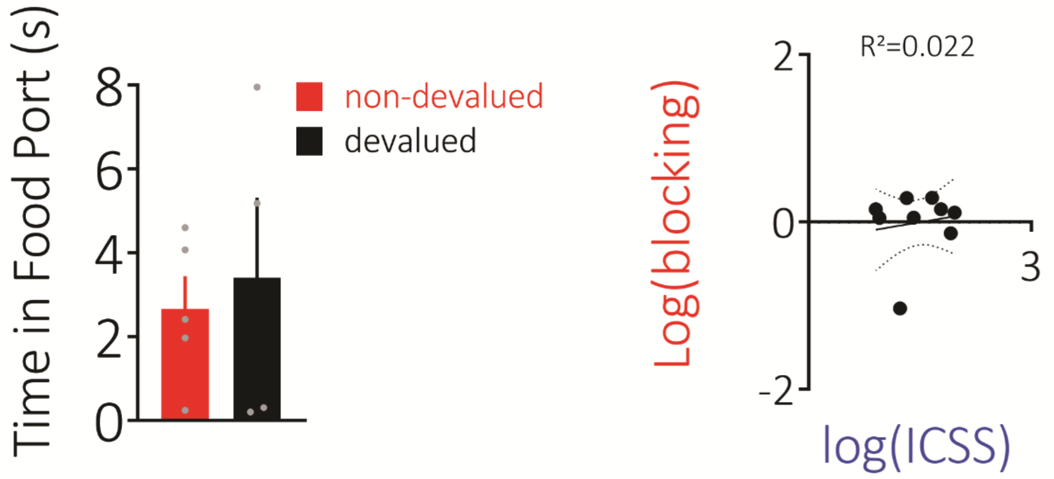

Fig S3. Responding to the blocked cue is not sensitive to devaluation and is not correlated with ICSS. Left: After devaluation of the reward paired with the unblocked cue, we tested responding to the blocked cue. We found that devaluation of the reward did not impact on responding to the blocked cue (one-tailed t-test, $F_{1,7}$=0.157, p=0.352). Right: Responding to the blocked cue in the initial probe test illustrated in the main text in Figure 3B did not correlate with the degree to which rats would press the active lever to get stimulation of dopamine neurons (Pearson's r=0.148, R^2=0.022; p=0.352). This experiment was not replicated in the current manuscript but reflects results similar to prior reports[3]. Error bars =SEM.

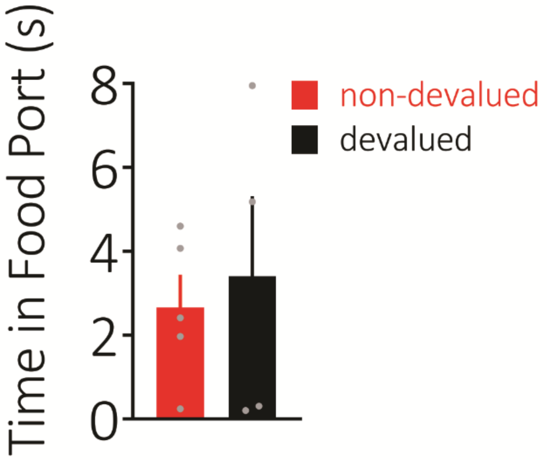

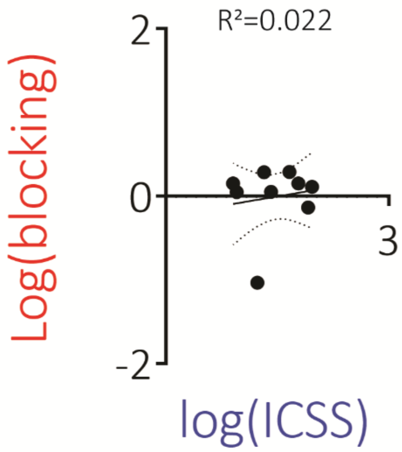

Fig S4. A physiologically-relevant frequency of dopamine stimulation (20Hz) functions as a prediction error to unblock learning of cue-reward associations. Top: schematic illustrating the task design. Bottom: food-port entries during conditioning (A), blocking (B), and the test (C). To ensure that our 20Hz stimulation was functioning to produce a physiologically-relevant signal, we tested whether these stimulation parameters would be sufficient to drive learning, as we and others have demonstrated previously. This experiment was conducted on the same rats that underwent the PIT experiment represented in Figure 2 of the main text. A) We trained rats (*n*=10) that a light led to delivery of two food pellets. All rats acquired the food-port entry response, with no difference between the groups (Repeated measures ANOVA, session: $F_{7,56}$=14.369, p=0.0001; session x group: $F_{7,56}$=0.344, p=0.930; group: $F_{1,8}$=0.119, p=0.739). B) Then, we presented a tone in compound with the light and the same food pellets. Usually, rats do not learn about the tone. However, when the food pellets were presented after the tone + light compound, we stimulated dopamine neurons at 20Hz in half the rats. All rats continued to exhibit high rates of responding at the food port, with no visible changes in these rates produced by dopamine stimulation (Repeated measures ANOVA, session: $F_{2,16}$=1.098, p=0.357; session x group interaction: $F_{2,16}$=1.608, p=0.218; group: $F_{1,8}$= 0.001, p=0.978). We then tested responding to the tone alone and found that dopamine stimulation had successfully unblocked learning (Repeated measures ANOVA, time: $F_{4,32}$=4.144, p=0.008; time x group: $F_{4,32}$=2.228, p=0.044; group (early): $F_{1,8}$=7.538, p=0.025), confirming our stimulation parameters were producing a physiologically-relevant dopamine signal. This experiment is a replication of the experiment presented in Figure C-D. Error bars = SEM. **indicates p<0.05.

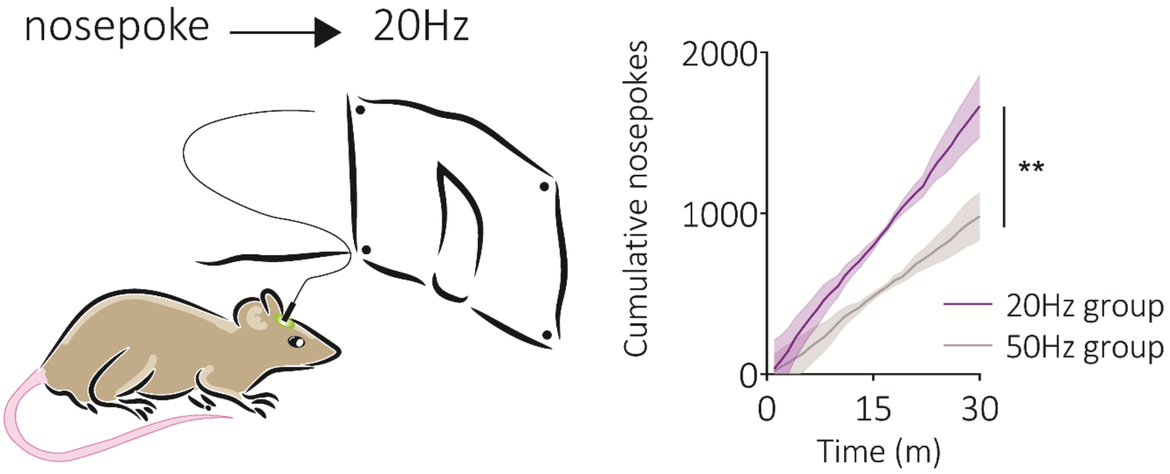

Fig. S5. Rats will nosepoke robustly for 20Hz stimulation of dopamine neurons on a continuously-reinforced schedule. We tested whether we could replicate the finding that rats will nosepoke at high rates for 20Hz dopamine stimulation[17]. To do this, we took the rats that had experienced the PIT procedure illustrated in Fig 1 of the main text and examined how much they would nosepoke for a 1s 20Hz train of stimulation on a continuously reinforced schedule. Consistent with prior publications[17], we found that the rats that had previously received 20Hz of dopamine stimulation would nose poke robustly for 20Hz dopamine stimulation across the session. This shows that our optogenetic preparation was working well and we could generate high levels of ICSS using the 20Hz stimulation train under low effort procedures. However, rats that had previously received 50Hz of dopamine stimulation would nose poke for 20Hz, but significantly less than the 20Hz group. This was confirmed with a repeated-measures ANOVA on the cumulative data, which showed a main effect of time ($F_{29,232}$=30.830, p=0.000), and an interaction by group ($F_{29,232}$=1.798, p=0.010), demonstrating that our 20Hz group increased responding across time at a greater rate than the 50Hz group. This could suggest that reducing the frequency of dopamine stimulation from 50 to 20Hz devalued the stimulation. Alternatively, this could be due to differential changes in dopamine circuits resulting from chronic 20Hz and 50Hz stimulation during the PIT procedures. This experiment is a replication of a previous report[17]. ****indicates p<0.05.
