# Supplementary material for: Cognitive representations of intracranial self-stimulation of midbrain dopamine neurons depend on stimulation frequency
Source: Nat Neurosci. 2024 May 13;27(7):1253–9. doi: 10.1038/s41593-024-01643-1 (PMC11239488; doi:10.1038/s41593-024-01643-1)
Supplement: Supplementary file 2 — Reporting Summary [file 41593_2024_1643_MOESM2_ESM.pdf]

nature portfolio

Corresponding author(s): Melissa Sharpe

Last updated by author(s): 03/22/2024

Reporting Summary

Nature Portfolio wishes to improve the reproducibility of the work that we publish. This form provides structure for consistency and transparency in reporting. For further information on Nature Portfolio policies, see our Editorial Policies and the Editorial Policy Checklist.

Statistics

For all statistical analyses, confirm that the following items are present in the figure legend, table legend, main text, or Methods section.

n/a	Confirmed	
☐	☒	The exact sample size (*n*) for each experimental group/condition, given as a discrete number and unit of measurement
☐	☒	A statement on whether measurements were taken from distinct samples or whether the same sample was measured repeatedly
☐	☒	The statistical test(s) used AND whether they are one- or two-sided<br>*Only common tests should be described solely by name; describe more complex techniques in the Methods section.*
☒	☐	A description of all covariates tested
☐	☒	A description of any assumptions or corrections, such as tests of normality and adjustment for multiple comparisons
☐	☒	A full description of the statistical parameters including central tendency (e.g. means) or other basic estimates (e.g. regression coefficient) AND variation (e.g. standard deviation) or associated estimates of uncertainty (e.g. confidence intervals)
☐	☒	For null hypothesis testing, the test statistic (e.g. *F*, *t*, *r*) with confidence intervals, effect sizes, degrees of freedom and *P* value noted<br>*Give P values as exact values whenever suitable.*
☒	☐	For Bayesian analysis, information on the choice of priors and Markov chain Monte Carlo settings
☒	☐	For hierarchical and complex designs, identification of the appropriate level for tests and full reporting of outcomes
☐	☒	Estimates of effect sizes (e.g. Cohen's *d*, Pearson's *r*), indicating how they were calculated

Our web collection on statistics for biologists contains articles on many of the points above.

Software and code

Policy information about availability of computer code

Data collection	Behavioral Data was collected using Med Associates software, which allowed us to automate data collection.
Data analysis	Med Associates v5 software produced output that was then transferred using MEDPC2XL. SPSS v29 was then used to conduct appropriate statistical analyses on the processed data. For fiber photometry analyses, we used MATLAB 2023a to process data using custom code.

For manuscripts utilizing custom algorithms or software that are central to the research but not yet described in published literature, software must be made available to editors and reviewers. We strongly encourage code deposition in a community repository (e.g. GitHub). See the Nature Portfolio guidelines for submitting code & software for further information.

Data

Policy information about availability of data

All manuscripts must include a data availability statement. This statement should provide the following information, where applicable:

- Accession codes, unique identifiers, or web links for publicly available datasets
- A description of any restrictions on data availability
- For clinical datasets or third party data, please ensure that the statement adheres to our policy

Data are available upon request to melissa.sharpe@sydney.edu.au

Human research participants

Policy information about studies involving human research participants and Sex and Gender in Research.

Reporting on sex and gender	N/A
Population characteristics	N/A
Recruitment	N/A
Ethics oversight	N/A

Note that full information on the approval of the study protocol must also be provided in the manuscript.

Field-specific reporting

Please select the one below that is the best fit for your research. If you are not sure, read the appropriate sections before making your selection.

☐ Life sciences ☒ Behavioural & social sciences ☐ Ecological, evolutionary & environmental sciences

For a reference copy of the document with all sections, see nature.com/documents/nr-reporting-summary-flat.pdf

Behavioural & social sciences study design

All studies must disclose on these points even when the disclosure is negative.

Study description	We used optogenetics and neural recordings to determine how different frequencies of dopamine neuronal stimulation act to motivate behavior. All data collected are quantitative, with the exception of histological analyses.
Research sample	Our rats are taken from our breeding colony of TH-Cre rats, a transgenic line purchased from RRRC and bred with Charles River wild type rats. We use males and females at around 3 months of age. This allows us to specifically manipulate dopamine neurons.
Sampling strategy	All groups are matched by age and sex. We chose sample sizes on the basis of previous work (e.g., Sharpe et al. 2016, NPP). Post-hoc power analyses were also performed for results in Figure 1 and 3H-N, with power>0.8.
Data collection	All data are collected with automated software described in the text, with the exception of histological analyses. In the case of histological analysis, experimenters are not blind to group allocations.
Timing	Data were collected across a period including 06/06/2019-10/10/2023.
Data exclusions	We excluded one rat from the experiment represented in Supp Fig 2 as they exhibited behavior 2 SDs beyond the group mean.
Non-participation	No human participants were used in this study.
Randomization	All rats are randomly allocated to groups, which are matched by sex and age.

Reporting for specific materials, systems and methods

We require information from authors about some types of materials, experimental systems and methods used in many studies. Here, indicate whether each material, system or method listed is relevant to your study. If you are not sure if a list item applies to your research, read the appropriate section before selecting a response.

Materials & experimental systems

n/a	Involved in the study
☐	☒ Antibodies
☒	☐ Eukaryotic cell lines
☒	☐ Palaeontology and archaeology
☐	☒ Animals and other organisms
☒	☐ Clinical data
☒	☐ Dual use research of concern

Methods

n/a	Involved in the study
☒	☐ ChIP-seq
☒	☐ Flow cytometry
☒	☐ MRI-based neuroimaging

Antibodies

Antibodies used	Red (594) antibodies: abbit anti-TH; Sigma-Aldrich, MO; goat anti-rabbit IgG Alexa Fluor 594; ThermoFisher Scientific, MA
Validation	These antibodies are purchased from Thermofisher and Sigma-Aldrich. Data on their successful validation experiments, and use of these antibodies have been cited in over 2000 research articles.

Animals and other research organisms

Policy information about studies involving animals; ARRIVE guidelines recommended for reporting animal research, and Sex and Gender in Research

Laboratory animals	Long Evans rats, around 3 months of age at the start of the experiment
Wild animals	No wild animals were used in this study
Reporting on sex	We used male and female rats in this study.
Field-collected samples	No field collected samples were used in this study.
Ethics oversight	Studies were in accordance with Animal Research Council at UCLA and Animal Care and Use Committee at Rutgers University.

Note that full information on the approval of the study protocol must also be provided in the manuscript.
